# OncoThreads: visualization of large-scale longitudinal cancer molecular data

**DOI:** 10.1093/bioinformatics/btab289

**Published:** 2021-07-12

**Authors:** Theresa A Harbig, Sabrina Nusrat, Tali Mazor, Qianwen Wang, Alexander Thomson, Hans Bitter, Ethan Cerami, Nils Gehlenborg

**Affiliations:** Department of Biomedical Informatics, Harvard Medical School, Boston, MA 02115, USA; Department of Biomedical Informatics, Harvard Medical School, Boston, MA 02115, USA; Department of Data Science, Dana-Farber Cancer Institute, Boston, MA 02215, USA; Department of Biomedical Informatics, Harvard Medical School, Boston, MA 02115, USA; Oncology Bioinformatics, Novartis Institutes for BioMedical Research, Cambridge, MA 02139, USA; Oncology Bioinformatics, Novartis Institutes for BioMedical Research, Cambridge, MA 02139, USA; Department of Data Science, Dana-Farber Cancer Institute, Boston, MA 02215, USA; Department of Biomedical Informatics, Harvard Medical School, Boston, MA 02115, USA

## Abstract

**Motivation:**

Molecular profiling of patient tumors and liquid biopsies over time with next-generation sequencing technologies and new immuno-profile assays are becoming part of standard research and clinical practice. With the wealth of new longitudinal data, there is a critical need for visualizations for cancer researchers to explore and interpret temporal patterns not just in a single patient but across cohorts.

**Results:**

To address this need we developed *OncoThreads*, a tool for the visualization of longitudinal clinical and cancer genomics and other molecular data in patient cohorts. The tool visualizes patient cohorts as temporal heatmaps and Sankey diagrams that support the interactive exploration and ranking of a wide range of clinical and molecular features. This allows analysts to discover temporal patterns in longitudinal data, such as the impact of mutations on response to a treatment, for example, emergence of resistant clones. We demonstrate the functionality of *OncoThreads* using a cohort of 23 glioma patients sampled at 2–4 timepoints.

**Availability and implementation:**

Freely available at http://oncothreads.gehlenborglab.org. Implemented in Java Script using the cBioPortal web API as a backend.

**Supplementary information:**

Supplementary data are available at *Bioinformatics* online.

## 1 Introduction

New profiling technologies, including next-generation sequencing, have significantly expanded our molecular understanding of cancer. Projects such as The Cancer Genome Atlas, the International Cancer Genome Consortium and the Human Tumor Atlas Network have set out to comprehensively characterize tumor samples by generating multi-omic datasets which support the identification of molecular subtypes and new, targeted treatment opportunities (Rozenblatt-Rosen *et al.*, 2020; The International Cancer Genome Consortium, 2010; Tomczak *et al.*, 2015).

These projects have sparked the development of new tools to visualize and explore these large datasets, including the cBioPortal for Cancer Genomics, a widely used platform for the analysis and visual exploration of cancer genomic datasets (Cerami *et al.*, 2012; Gao *et al.*, 2013); genomic browsers like UCSC Xena (Goldman *et al.*, 2020) and others (Nusrat *et al.*, 2019); and cohort visualization tools like StratomeX (Kern *et al.*, 2017; Lex *et al.*, 2012; Streit *et al.*, 2014).

Despite the advancement of cancer-specific visualizations and portals, temporal visualizations are often lacking. cBioPortal offers a temporal view for individual patients which supports a range of data types, including procedure and treatments (Cerami *et al.*, 2012; Gao *et al.*, 2013). Another temporal visualization is the ‘fishplot’, which shows the development of tumor subclones in an individual over time (Dang *et al.*, 2017; Miller *et al.*, 2016). However, neither approach scales well for entire cohorts, as subclone evolution is highly individual and cohort visualizations with individual patient timelines become cluttered even for a small number of patients and time points. Tools like EventFlow (Monroe *et al.*, 2013) and DecisionFlow (Gotz and Stavropoulos, 2014) tackle this problem by aligning shared events in cohorts in blocks with transitions between events displayed as flows. Another approach has been implemented by Perer and Sun (2012), where events in a cohort are grouped into timepoints and displayed in matrices showing the co-occurrence of events. While these approaches are useful for analyzing event sequences, as well as for selecting and comparing cohorts (Malik *et al.*, 2015), they do not integrate multiple features for events, such as mutation data and expression data for sample collection events. A more flexible block-based technique is Domino, which is a visualization technique for the creation of multiple connected visualizations (Gratzl *et al.*, 2014). Despite not being developed specifically for temporal data, a wide range of temporal visualizations can be implemented and modified directly in the tool. However, due to its high flexibility and the novel underlying concept, it is difficult to apply for users who are not visualization experts.

*OncoThreads* was designed for cancer researchers and developed to address the lack of temporal cohort visualization tools, which specifically integrate multiple molecular data types and clinical data. *OncoThreads* provides exploratory visualizations of longitudinal cancer molecular data across patient cohorts and supports a wide range of biological data types, including mutations, copy number alterations, mRNA expression and protein expression. Furthermore, *OncoThreads* offers a temporal cohort visualization based on heatmaps and Sankey diagrams as well as a timeline overview for all patients. Moreover, it provides a feature explorer to discover features of interest—variables that are defined for each patient and timepoint, such as tumor stage or mutation burden—and feature modification in order to adjust their visual representation and facilitate interpretation. We demonstrate the ability of *OncoThreads* to enable the exploration of longitudinal cancer molecular data in a comprehensive case study with a cohort of 23 glioma patients (Section 3, also Supplementary Video and Figures). Moreover, we assess the usefulness of the design sprint approach (Knapp *et al.*, 2016) for the development of exploratory visualizations.

## 2 Materials and methods

### 2.1 *OncoThreads* overview

**Fig. 1.**
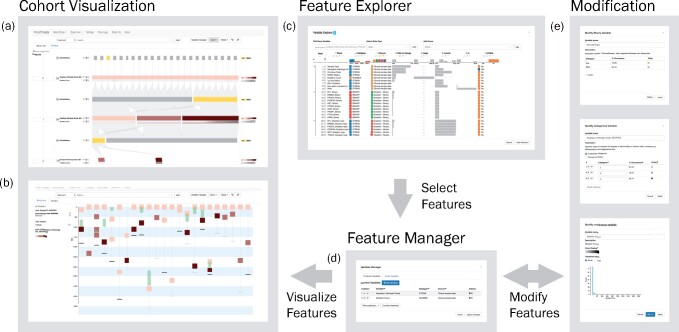
A schematic view of the components of *OncoThreads*. Molecular data can be visualized in two separate views, the block view (**a**), which aligns shared events of patients as blocks and the timeline view (**b**), which shows a timeline for each patient. Features of interest can be found and selected in the Feature Explorer (**c**) and added to the Feature Manager (**d**), which supplies them to the visualization. The application also enables feature modification using different types of transformations depending on the type of the feature (**e**)

*OncoThreads* enables researchers to dynamically visualize longitudinal clinical and molecular data across an entire patient cohort, allowing for the identification of patterns in cancer evolution. For example, researchers can visualize tumor stage, mutations, mRNA expression levels or tumor mutation burden at multiple timepoints for an entire patient cohort. The application consists of several components for the selection of features and temporal visualization (Fig. 1).

*OncoThreads* displays time as a vertical flow from top to bottom in order to accommodate large patient cohorts, which are presented horizontally. The selected features can be visualized in two separate views. In the block view, samples and events are aligned in blocks in order to show general event patterns of the cohort over time (Fig. 1a). The timeline view shows a timeline for each patient reflecting the actual temporal distance between samples and events (Fig. 1b). A user can alternate between these two views as data are explored. In order to keep track of the exploration, every action is saved in an accessible log and undo/redo functionality is provided. Additionally, users can export the current view, including detailed metadata about the displayed features, in multiple file formats (PNG, PDF or SVG).

Data can either be loaded using the cBioPortal API or local files. With the Feature Explorer, features can be ranked and selected according to attributes, such as their variability over time (Fig. 1c). Additionally, features can be transformed in the Feature Manager, for example, to change a feature’s color scale or to convert a continuous feature to an ordinal feature by binning or to aggregate genes into gene sets (Fig. 1d and e).

### 2.2 Block view

The main visual element of the *OncoThreads* cohort visualization is a block. *OncoThreads* supports two types of blocks: timepoint blocks and event blocks. A timepoint block represents the samples of a patient cohort at a certain timepoint with associated clinical, genomic or other molecular data (e.g. samples acquired at initial and recurrent surgeries, or prior to and following a therapy). An event block represents events that occur between two timepoints (e.g. treatment with a drug). Timepoint blocks are always visible, while event blocks can be added as desired; when both are visible, event and timepoint blocks alternate (Fig. 1a and also see Section 3). The rows of a block represent a set of features. Upon loading a study, data within the blocks is visualized as a heatmap. Data within blocks can be rearranged to explore the data by sorting the entire heatmap with respect to a feature at a specific timepoint, or transforming it into a Sankey diagram by grouping.

**Fig. 2.**
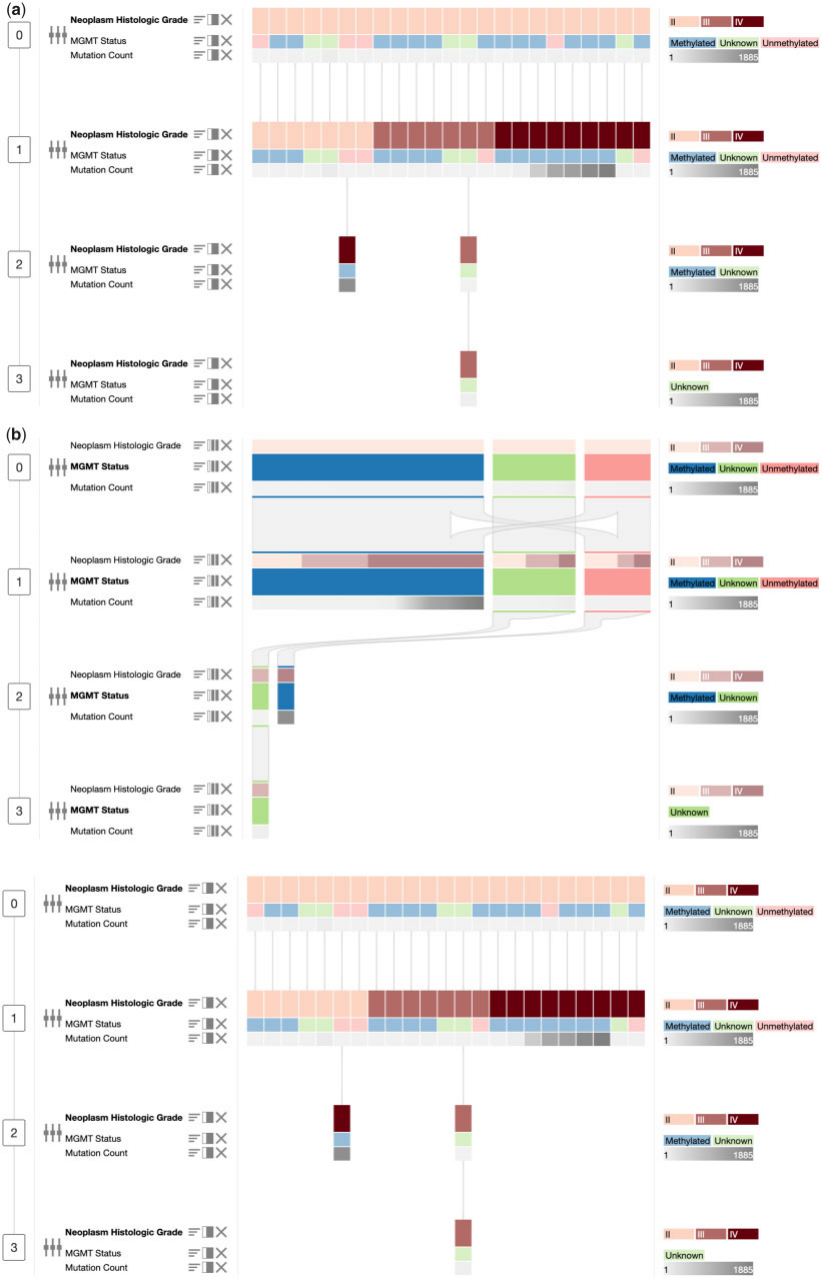
Visualization operations in *OncoThreads*. Blocks represent timepoints, which are ordered vertically. (**a**) Heatmap view with multiple sample-level clinical features (mutation count, MGMT status and neoplasm histologic grade). Patients are connected by lines. Multidimensional sorting is applied to the second timepoint, which is primarily sorted by neoplasm histologic grade, while the secondary order is given by the other features. Patients in the other blocks have been aligned based on the order of this timepoint. (**b**) The same data with all blocks grouped by MGMT status. Grouped blocks show proportions of patients instead of single patients. Within the primary grouping (MGMT status) the other features are grouped as well. Bands show the proportion of patients transitioning between feature values of two blocks

Sorting enables the exploration of the distribution of values of a feature. Each block can be sorted individually with respect to a feature (called the primary feature). Since sorting may change the order of the patients to be different across timepoints, the connecting lines are curved and may cross. In order to eliminate crossing lines, the patients can be realigned with respect to the patient order in any of the blocks (Fig. 2a). Moreover, we also implemented multidimensional sorting, which sorts based on multiple features at once. When a block is sorted repeatedly by different features, the previous order of patients is retained and applied in case of ambiguities. This can be seen in Figure 2a, where the second timepoint is sorted by all three features.

The block view visualization can be transformed iteratively into a Sankey diagram by grouping timepoints to analyze the data as groups of patients rather than individual patients (Fig. 2b). A grouped block shows information about the proportions of patients based on the primary feature, rather than showing individual patient data (see also the case study in Section 3 and Supplementary Video). It therefore represents an aggregated view, while the heatmap shows the data in more detail. Due to the independent grouping and ungrouping of blocks, detail can be viewed selectively for certain timepoints, while others stay grouped and show proportions. Furthermore, grouping is especially useful for large cohorts since it might not be possible to visualize the entire cohort as a heatmap depending on the screen width.

If the primary feature is categorical, the proportions in grouped blocks are displayed as horizontal bars with widths corresponding to the size of the proportion. The proportions and distributions of other features are shown within the groupings of the primary feature to allow a comprehensive comparison of the compositions of different grouped blocks. Values of continuous features are summarized by visualizing their distributions using color gradients or boxplots. For continuous features many patients have unique values, which would lead to one patient per group. Consequently, a continuous feature has to be binned before grouping to transform it into a categorical feature as described in Section 2.4.1. A Sankey diagram is created whenever two adjacent blocks are grouped. The connection between blocks changes to bands showing the fraction of patients transitioning between the proportions of the blocks. To highlight that the bands originate from the primary feature and not from the last row of the grouped block, the colors of the primary feature are repeated as a proxy at both ends of the connections (Fig. 2b).

By default, patient samples are aligned with the first available timepoint for each patient as the first timepoint in the visualization. However, a cohort may have variability in the first available timepoint, or it may be of interest to analyze a cohort relative to an event instead, such as the administration of a treatment. Therefore, we implemented flexible timepoint alignment. Patient columns can be selected in an ungrouped block and moved up or down using a context menu. Section 3 shows how this functionality is applied in a sample dataset.

In order to track a subset of patients in the visualization, *OncoThreads* allows a user to select individual patients as well as groups of patients. The selected patients or patient groups are highlighted in all blocks and bands allowing the user to gain an understanding of the composition of a subset of patients in all blocks simultaneously.

### 2.3 Timeline view

In the timeline view, data are visualized as a series of adjacent vertical timelines, one timeline for each patient. Users can switch between the block view and the timeline view to analyze different aspects of the data. The timeline view can address questions such as the relationship between the duration of a therapy and time to progression. In this view, only one feature is displayed for each sample. Different events are encoded using different colors, and the duration of an event is encoded by the length of the bar (Fig. 1b). Similar to the block view, patients can be selected interactively. Selected patients are retained in both views. Therefore, patients can be analyzed as an aligned cohort in the block view and their temporal patterns can be viewed by switching to the timeline view.

### 2.4 Feature operations

*OncoThreads* supports a wide range of data types, including gene-specific data like mutations or expression as well as clinical data, which may be timepoint- and patient-specific or just patient-specific. Clinical data are pre-loaded upon study selection, while gene-specific data are queried on-demand using the HUGO gene symbol and the datatype of interest. *OncoThreads* includes a Feature Manager to transform features and change the order of currently displayed features. Additionally, a Feature Explorer is provided for the discovery of features to be added to the visualization via guided exploration (Streit *et al.*, 2014). For convenience, known features of interest can also be selected using a drop-down menu in the toolbar of the visualization.

#### 2.4.1 Feature Manager

Features are added to the view exactly as the data are provided, which may not be optimal for visualization. For example, application of a log scale might enhance the interpretation of continuous data with a wide range of values or combining multiple genetic features can enable pathway-based analysis. Therefore, the Feature Manager enables users to transform features (Fig. 1d and e). All currently displayed features can be modified. Continuous features can be log transformed or binned to transform them to an ordinal feature, categorical features can be converted to ordinal features and vice versa, and binary features can be inverted. Moreover, features of the same kind can be combined. For example, binary features encoding for the presence of mutations in specific genes can be combined using a Boolean operator in order to quickly identify patients or groups of patients showing a combination of these mutations. In addition, every feature can be renamed and the color scale can be changed. The Feature Manager also enables changing the order of the features in the view, either manually or through sorting by an attribute like datatype, source (clinical, expression, mutation, etc.) or name. In Section 3 and in the Supplementary Video, we demonstrate the usage of the feature operations in a case study.

#### 2.4.2 Feature Explorer

**Fig. 3.**
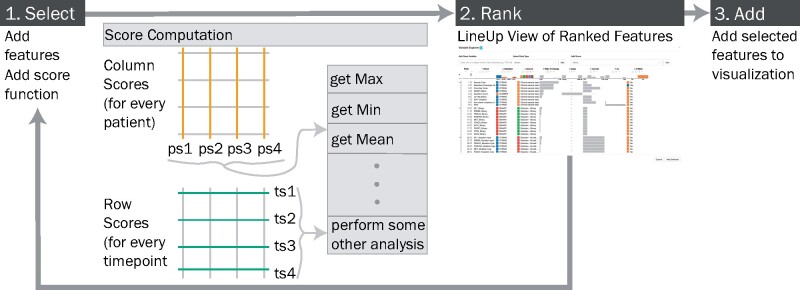
The framework shows four basic steps for feature exploration. (1) Column scores and row scores can be selected to assess both variability within timepoints and across timepoints for each feature in the Feature Explorer. Scores are calculated for each timepoint or patient and aggregated using a method of choice (grey box). (2) Features can be ranked by the calculated scores using LineUp (Gratzl *et al.*, 2013). (3) Features of interest can be selected in LineUp and added to the visualization

The Feature Explorer supports guided exploration and selection of features (Fig. 1c). It provides an overview of all clinical features and any genomic or molecular features that have been added, including range for continuous features, or data types, data source, etc. In addition, the Feature Explorer provides variability scores to highlight features that may be of biological interest due to high variability within a timepoint or across timepoints. These scores are measures of statistical dispersion that indicate the extent to which a distribution is stretched or squeezed. Users can select different scores using a drop-down menu and can see the ranking of every feature based on these scores (Fig. 3). This ranking is shown with an interactive technique called LineUp (Gratzl *et al.*, 2013) which helps users prioritize features, evaluate them and understand any correlations among them. Similarly to StratomeX (Lex *et al.*, 2012; Streit *et al.*, 2014), features can be selected in LineUp and added to the visualization.

**Fig. 4.**
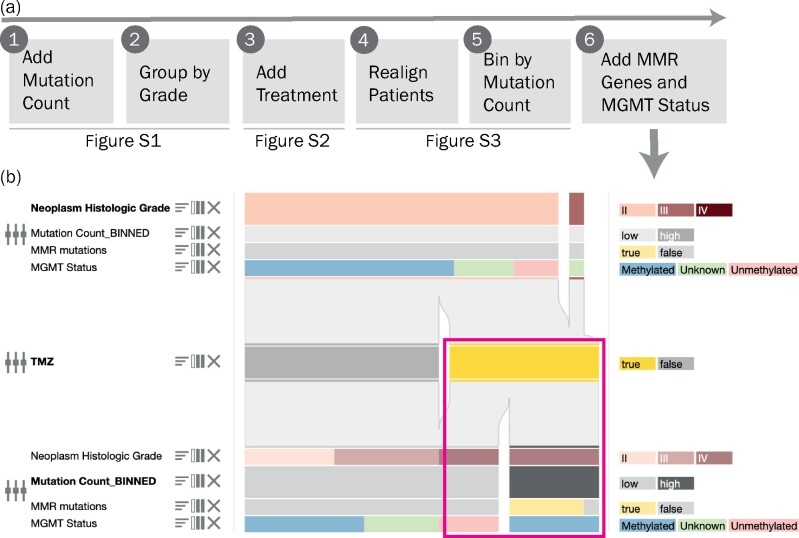
The examples in (**a**) and (**c**) show high variability within a timepoint, but no variability across timepoints for categorical and numerical data. Examples in (**b**) and (**d**) show the opposite pattern

We examine two types of variability of features in *OncoThreads*: within timepoint and across timepoints (Fig. 4). Variability within a timepoint examines how consistent the data for a feature is across all patients at each timepoint. Variability across timepoints examines how a feature changes over time for individual patients. Figure 4a shows data with high within timepoint variability, but low variability across timepoints. In contrast, Figure 4b shows low variability within timepoints and high variability across timepoints. A similar concept can be applied for numerical data (Fig. 4c and d). However, different methods are required to calculate variability scores for the different data types.

Variability scores can be calculated both within timepoints (row scores) and across timepoints (column scores). We can aggregate these scores to obtain a single score for every feature. For example, consider a feature in four timepoints. We can calculate variability scores for this feature for each timepoint. These scores can then be aggregated to a single score by selecting the maximum, minimum or average of the four timepoint scores (Fig. 3). Scores for all features can be compared within the Feature Explorer, allowing a user to rank features and find correlations among them. ModVR measures variation around the mode (Wilcox, 1973). It is a standardized form of the variation ratio, a measure of statistical dispersion in nominal data, or the proportion of cases that are not in the ‘mode’ category. The ModVR values range from 0, indicating low variability, to 1, indicating high variability. The coefficient of unalikeability measures variability for categorical data. It represents the proportion of observations that differ. The higher the value, the more unalike the data are (Kader and Perry, 2007). The coefficient of variation (CV) is the ratio of the standard deviation to the mean. A CV <1 indicates low variance, whereas a CV >1 indicates high variance. For categorical features the rate of change is the number of values that changed relative to the total number of value transitions. For continuous features it represents the rate of the average change to the observed range. Developers can implement additional scores for this extensible ranking framework.

### 2.5 Design process

We employed the design sprint methodology (Knapp *et al.*, 2016) to enable our multi-institutional team to develop consensus goals as well as to obtain user feedback prior to undertaking a full development and implementation process. We also set out to evaluate the success of applying a design sprint to visualization problems. We performed the design sprint with a group of six people with backgrounds in biology, biomedical informatics and visualization over five consecutive days, for 6 h each day. The overall goal for our effort was to ‘develop the “go-to” visualization approach for longitudinal cancer molecular data through an agile framework that will have measurable technical and scientific impact’.

As part of the process, we interviewed three cancer researchers for 30 min each in addition to the authors to identify the most important challenges that needed to be addressed, which raised questions such as ‘How might we visualize an entire cohort over time?’; ‘How might we integrate multiple data types into one visualization?’; ‘How might we define timepoints?’; and ‘How might we enable the flexible analysis of a cohort relative to any event, for example, diagnosis or treatment?’

We examined existing tools and visualization strategies, including StratomeX (Lex *et al.*, 2012), Domino (Gratzl *et al.*, 2014), streamgraphs and Sankey diagrams; these inspired sketches from which we decided to utilize heatmaps and Sankey diagrams as the core components of the visualization. The visualization consists of connected blocks with the rows representing multiple features at different points in time. In order to facilitate finding patterns in the data, users can switch between the heatmap and the Sankey diagram as well as sort the visualization by a chosen feature. We reviewed an existing cancer evolution study (Johnson *et al.*, 2014) and used one of its main findings to define a path through the data which we could implement as a prototype of linked slides with the presentation software Apple Keynote. Given the time constraints of the design sprint, the prototype allowed for just a single path, rather than all possible paths of exploration.

We tested the prototype with four cancer researchers, all of whom successfully arrived at the scientific conclusion that we intended and found the tool useful overall. However, users also identified many opportunities for improvement; the primary issues were that users struggled due to the limited interactivity of the prototype and that the Sankey visualization in the prototype was confusing and did not provide an advantage over the heatmap.

Based on the feedback we received, we made two major changes to the concept: (i) instead of sorting the whole visualization by a single feature, we enabled independent sorting for each block, and similarly (ii) transform from a heatmap to a Sankey diagram iteratively by grouping blocks individually. The independent sorting and grouping of blocks prevents the visualization from changing too quickly, which we identified as a potential reason for misinterpretation of the prototype visualization. Moreover, selectively viewing blocks in detail enhances the exploration by adding flexibility.

### 2.6 Availability and implementation

*OncoThreads* is a web application available at http://oncothreads.gehlenborglab.org and its source code is available at https://github.com/hms-dbmi/oncothreads under the MIT license. *OncoThreads* is implemented in JavaScript using the libraries React (https://reactjs.org/), mobx (https://mobx.js.org) and D3 (https://d3js.org/) (Bostock *et al.*, 2011) for the application structure, state management and visualization, respectively. React-bootstrap (https://react-bootstrap.github.io) has been used to apply bootstrap styles to the React components. We retrieve data from the cBioPortal using their REST (Representational State Transfer) API with the promise-based library axios (https://github.com/axios/axios). Additionally, *OncoThreads* can be obtained as an Electron app (https://electronjs.org) available for download at https://github.com/hms-dbmi/oncothreads/releases.

## 3 Results: case study in low-grade glioma cohort

In a study by Johnson *et al.* (2014), the authors explored the genomic evolution of low-grade glioma by analyzing a cohort of 23 patients with samples from an initial resection as well as one or more recurrences. Samples were profiled with whole-exome sequencing and patients were clinically annotated. Among the findings of the paper was the impact of the chemotherapy temozolomide (TMZ) on low-grade gliomas; in six patients, tumor samples acquired after treatment with TMZ showed hypermutation and progression to high-grade glioblastoma in the context of MGMT silencing and loss of mismatch repair.

**Fig. 5.**
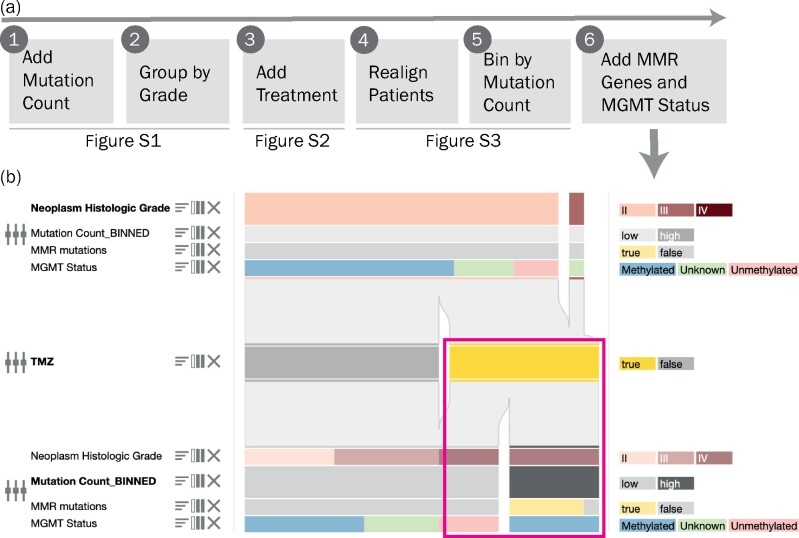
Exploring the glioma dataset of a study by Johnson *et al.* (**a**) Overview of the exploration. (**b**) Detailed view of the final step. Time is vertical, and two timepoints are shown. We can observe that most patients are classified as grade II at timepoint 1, and that most patients progress to grade III or IV at timepoint 2. Furthermore, all patients with a high mutation count received prior TMZ treatment and methylation of MGMT and mutations in mismatch repair genes co-occur with high mutation count (magenta box)

Figure 5a illustrates specific steps in an exploration of the data from Johnson *et al.* that demonstrates how the features of *OncoThreads* support the discovery of relevant subgroups within the patient cohort. After selection of the relevant dataset [Low-Grade Gliomas (UCSF, Science 2014)], a single feature, neoplasm histologic grade, is automatically rendered in the block view. By using the Feature Explorer and applying the Rate of Change score, we find that several features, including mutation count, show variability over time and are therefore especially interesting for analyzing differences between initial resection and recurrence. To explore the temporal patterns in more detail, we add mutation count and group both timepoints 1 and 2 by neoplasm histologic grade. This allows us to visualize specific trends in the data; for example, we observe that all patients have grade II tumors in the first timepoint block, but many develop a higher grade tumor at later timepoints. We also observe significantly increased mutation counts in grade IV tumors at timepoint 2 (Supplementary Fig. S1). We can now ask what factors may have influenced tumor development from grade II to grades III and IV.

In the Feature Manager we add TMZ treatment, and subsequently group the event block between timepoints 1 and 2 by TMZ treatment. We can then see that there is a notable flow from patients receiving TMZ to patients having a high grade in the second sample, suggesting that TMZ treatment may result in a higher grade recurrence (Supplementary Fig. S2). To further assess the effect of TMZ treatments for all patients, we realign the entire cohort relative to the treatment. We also want to see if the patients who received TMZ and developed a high-grade recurrence also have a high mutation count. Since mutation count is a continuous feature, we have to bin it first to transform it into a categorical feature as described in Section 2.4.1. Based on the distribution of mutations indicating that there are six samples exhibiting very high mutation counts, we create bins for low (<150 mutations) and high (≥150 mutations) mutation counts. Based on this exploration we can formulate the hypothesis that TMZ treatments correlate with high mutation count and grade IV at recurrence (Supplementary Fig. S3).

Given this correlation between TMZ treatment and increased mutational burden, we next look for additional evidence to functionally connect these two features. TMZ is a mutagen, and TMZ-induced mutations are believed to be mitigated by MGMT protein and the mismatch repair pathway (Liu and Gerson, 2006). Leveraging the available molecular data, we add additional tracks to show the mutational status of mismatch repair pathway genes MLH1, MSH6 and MSH3, and then use the Feature Manager to combine those tracks into a single track showing the overall mismatch repair pathway mutation status. We also add a track showing the MGMT methylation status of each sample. Now, examining those samples with high mutation count following TMZ treatment, we see that all samples show methylation of MGMT, indicating silencing of the gene and subsequent lack of protein, and almost all have mutations in mismatch repair pathway genes, which together support a potential causative role for TMZ in inducing hypermutation in these tumors (Fig. 5b).

## 4 Discussion

### 4.1 Application

The results of the case study demonstrate how the visual exploration features of *OncoThreads* support users in efficiently generating testable hypotheses and identifying supporting evidence through an effective combination of visualization and data integration tools. For example, *OncoThreads* helps researchers to explore the influence of a specific treatment on tumors in an entire patient cohort and to find patterns for the prediction of the outcome of a therapy. Furthermore, it may be used to discover patterns of genetic predispositions that can affect the effectiveness of a drug or help analyze the effects of different drug dosages.

Currently, *OncoThreads* utilizes variation around the mode (ModVR) for categorical data, and variance or CV for numerical data (Evren and Ustaoğlu, 2017) to rank features based on variability (Fig. 1c). However, these variability scores are implemented in an easily extensible framework, such that additional scores or aggregation approaches can be added, for example, calculating the variability score of a single timepoint rather than the aggregate across all timepoints to enable a query like ‘How do the features compare to each other based on their variability in timepoint 2?’

In the future, additional user interactions could trigger more complex queries in *OncoThreads*. An example of such a query could be: ‘Find all features that show a similar pattern in a specific timepoint’. Such a query would help users to identify correlations among features. In addition to queries involving sample features, event features could be taken into account in scoring functions to evaluate their relationship to sample features of subsequent timepoints. In general, these scoring mechanisms could guide users to features that provide additional insights and to generate new hypotheses.

With the undo and redo operation *OncoThreads* allows going back to previous steps during the exploration process. Yet, when a new action is performed after undoing, the previous path of exploration is lost. Therefore, it would be desirable to incorporate visualization provenance approaches such as Vistories (Gratzl *et al.*, 2016) or Trrack (Cutler *et al.*, 2020) into *OncoThreads*. In those approaches, the user’s actions are saved in a graph that captures all relevant interactions. Therefore, it is possible to go back to parts of the exploration that would be lost in regular undo/redo implementations. Moreover, those approaches allow the presentation of the results of the exploration by enabling the creation of a ‘replay’ that communicates the results by showing certain steps of the exploration with annotations.

In the future, we plan to improve scalability in the number of features and timepoints. One promising direction is to integrate sequential pattern mining and clustering techniques into the visual exploration of longitudinal patient data. These techniques can effectively learn patterns from complex sequential data and facilitate the identification of disease states. Moreover, we plan to enhance the representation of patient-specific data, as well as tumor heterogeneity. Although *OncoThreads* has been developed specifically for cancer data, it can also be applied to many other kinds of multidimensional temporal data.

### 4.2 Design sprint

To the best of our knowledge, the design sprint technique has not been documented for the development of a biomedical data visualization tool before. In the examples described by Knapp *et al.* (2016), the design sprint methodology is used for the development of tools and products without exploratory functionality. For example, when a website is designed for selling a product there are a few well-defined steps that a user has to conduct to purchase the product. In contrast, an explorative visualization can be used in many different ways and no clear endpoint is defined. Therefore, we recommend adapting the technique to visualization problems, especially to deal with the complexity of modeling their exploratory and interactive nature. For example, defining the workflow of the planned tool before conducting user interviews might introduce bias in the downstream process. It might be more useful to define required steps without specifying their order. Moreover, during prototyping, it is likely not feasible to implement all possible exploratory steps in the given timeframe, so we had to limit the exploration to one path. Similarly, time for sketching needs to be increased. Nevertheless, we found that the approach can be applied effectively to efficiently develop and test ideas despite the complexity of the data and to create a shared vision for the team. While the design sprint technique allowed us to get early feedback from users, a validation of *OncoThreads* with an insight-based evaluation approach (Saraiya *et al.*, 2005) could provide more information about the quality of the hypotheses generated with *OncoThreads*.

## Supplementary Material

btab289_Supplementary_Data
