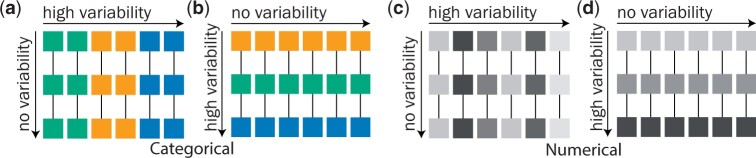# Correction to: OncoThreads: visualization of large-scale longitudinal cancer molecular data

**DOI:** 10.1093/bioinformatics/btae111

**Published:** 2024-03-04

This is a correction to: Theresa A Harbig, Sabrina Nusrat, Tali Mazor, Qianwen Wang, Alexander Thomson, Hans Bitter, Ethan Cerami, Nils Gehlenborg, OncoThreads: visualization of large-scale longitudinal cancer molecular data, Bioinformatics, Volume 37, Issue Supplement_1, July 2021, Pages i59–i66, https://doi.org/10.1093/bioinformatics/btab289

In the originally published version of this manuscript, the image of Figure 4 was incorrect and it duplicated the image of Figure 5.

The correct image for Figure 4 is given below:

These details have been corrected only in this correction notice to preserve the published version of record.

**Figure 4:**