# Supplementary material for: OncoThreads: visualization of large-scale longitudinal cancer molecular data
Source: Bioinformatics. 2021 Jul 12;37(Suppl 1):i59–66. doi: 10.1093/bioinformatics/btab289 (PMC8275328; doi:10.1093/bioinformatics/btab289)
Supplement: btab289_Supplementary_Data [file btab289_supplementary_data.zip › Harbig.73.sub/Harbig.73.sub.1.pdf]

Supplementary Figures

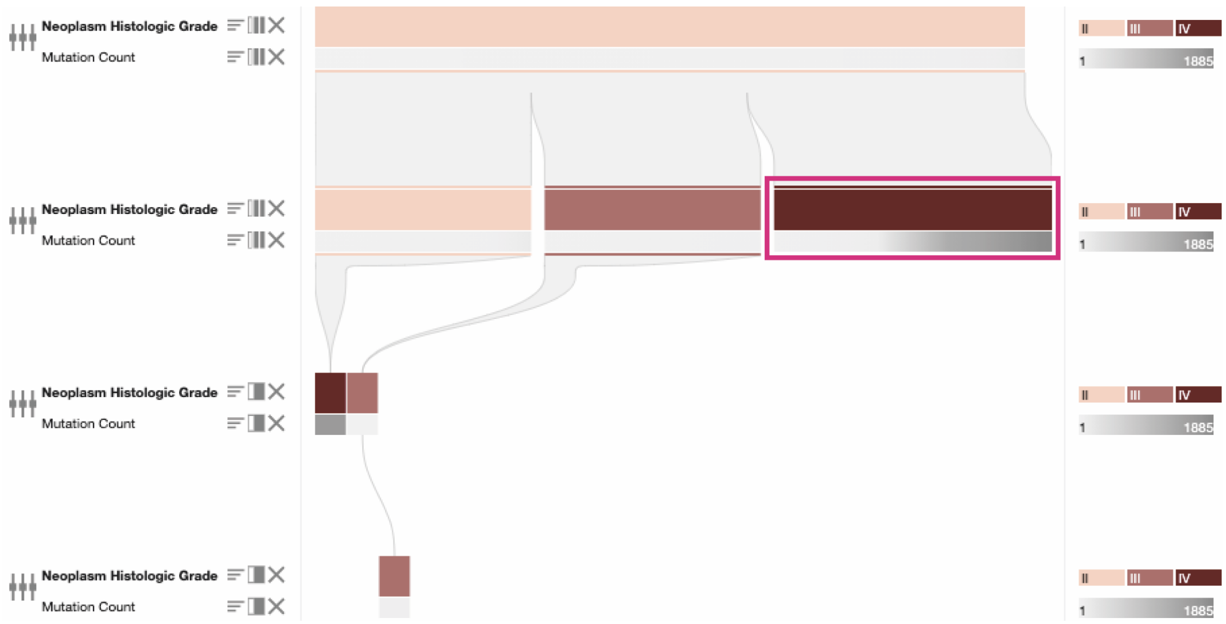

Figure S1: *First step of example analysis of glioma data using OncoThreads. Four time points are displayed vertically, along with two features at each time point: Neoplasm Histologic Grade and overall mutation count. Timepoints 1 and 2 are grouped by Neoplasm Histologic Grade. Visual inspection shows that all patients are grade II at timepoint 1, but many develop a higher grade tumor at later timepoints. Grade IV tumors at timepoint 2 are also associated with increased mutation rate (highlighted in magenta outline).*

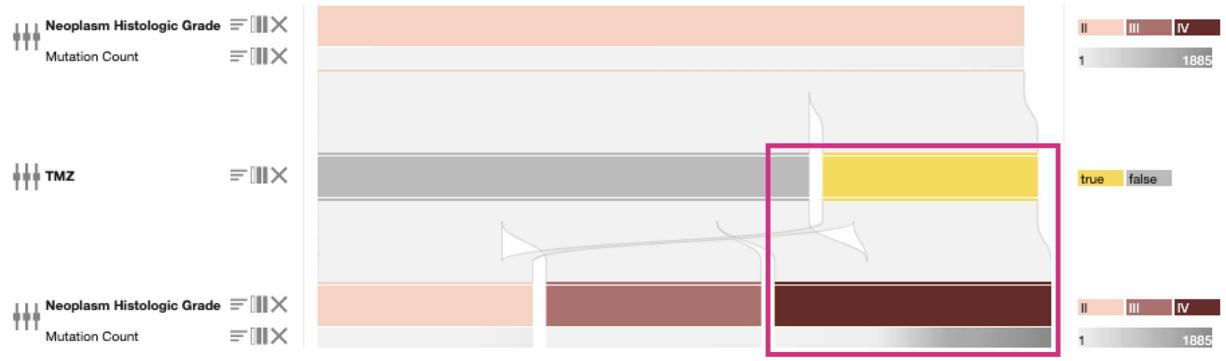

Figure S2: *Second step of an example analysis of glioma data using OncoThreads. TMZ treatment has been added and the event block between timepoints 1 and 2 has been grouped by TMZ. Most patients showing a high grade have been treated with TMZ (highlighted in magenta outline).*

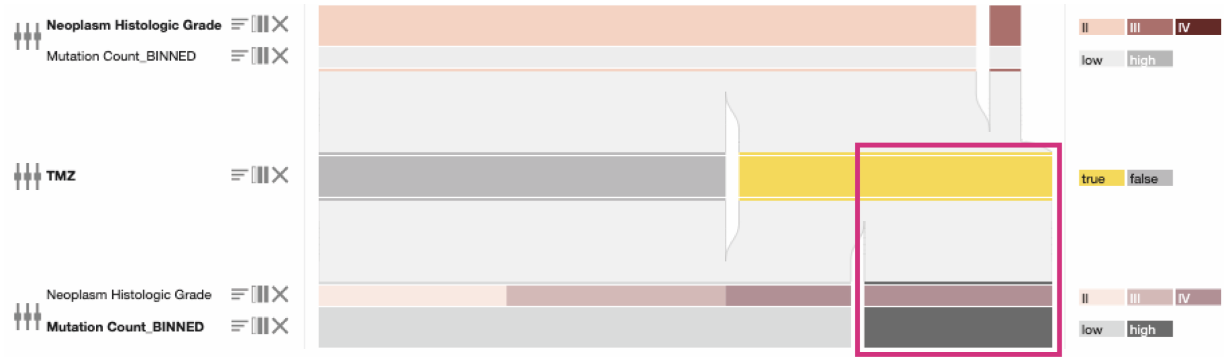

Figure S3: *Third step of an example analysis of glioma data using OncoThreads. Patients have been realigned based on TMZ treatment, and mutation count has been binned into high and low. All patients with a high mutation count were treated with TMZ and have a grade IV tumor (highlighted in magenta outline).*
